# Shell thickness dependent photostability studies of green-emitting “Giant” quantum dots†

**DOI:** 10.1039/d1na00663k

**Published:** 2021-09-24

**Authors:** Rahul Singh, Syed Akhil, V. G. Vasavi Dutt, Nimai Mishra

**Affiliations:** Department of Chemistry, SRM University-AP Amaravati, Neerukonda Guntur(Dt) Andhra Pradesh 522240 India nimai.m@srmap.edu.in

## Abstract

Highly efficient green-emitting core/shell giant quantum dots have been synthesized through a facile “one-pot” gradient alloy approach. Furthermore, an additional ZnS shell was grown using the “Successive Ionic Layer Adsorption and Reaction” (SILAR) method. Due to the faster reactivity of Cd and Se compared to an analogue of Zn and S precursors it is presumed that CdSe nuclei are initially formed as the core and gradient alloy shells simultaneously encapsulate the core in an energy-gradient manner and eventually thick ZnS shells were formed. Using this gradient alloy approach, we have synthesized four different sized green-emitting giant core–shell quantum dots to study their shell thickness-dependent photostability under continuous UV irradiation, and temperature-dependent PL properties of nanocrystals. There was a minimum effect of the UV light exposure on the photostability beyond a certain thickness of the shell. The QDs with a diameter of ≥8.5 nm show substantial improvement in photostability compared to QDs with a diameter ≤ 7.12 nm when continuously irradiated under strong UV light (8 W cm^−2^, 365 nm) for 48 h. The effect of temperature on the photoluminescence intensities was studied with respect to the shell thickness. There were no apparent changes in PL intensities observed for the QDs ≥ 8.5 nm, on the contrary, for example, QDs with <8.5 nm in diameter (for ∼7.12 nm) show a decrease in PL intensity at higher temperatures ∼ 90 °C. The synthesized green-emitting gradient alloy QDs with superior optical properties can be used for highly efficient green-emitters and are potentially applicable for the fabrication of green LEDs.

## Abstract 2

Green-emitting gradient alloy core/shell quantum dots with superior photostability under UV irradiation and high temperature.

## Graphical abstract

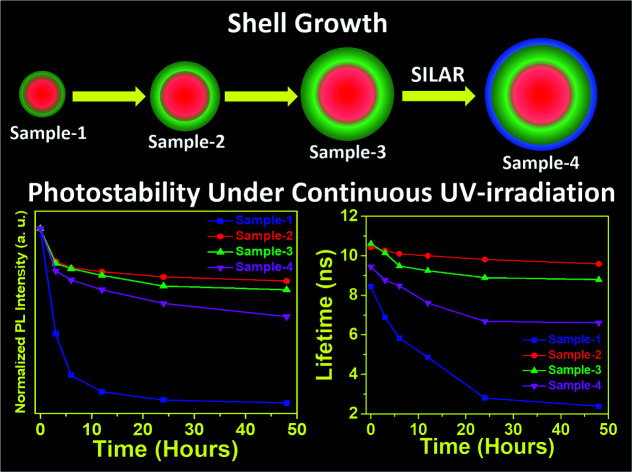

## Introduction

Colloidal semiconductor nanocrystals or colloidal quantum dots have been of great research interest due to their unique optical properties like broad absorption, and tunable and narrow emission.^1–3^ These optical properties make these colloidal nanocrystals suitable for different optoelectronic applications such as solar cells,^4–6^ light-emitting diodes (LEDs),^7,8^ photodetectors,^9,10^ lasing,^11,12^ and bio labelling.^13,14^ Among various compositions, CdSe colloidal quantum dots have been studied the most because of their excellent tunable optical properties covering the whole visible spectrum (400–700 nm).^3^ Thus, CdSe becomes an ideal candidate for several optoelectronic and biological applications. However the surface of bare CdSe quantum dots suffers from oxidation and photodegradation under ambient conditions, thus hindering its possibilities for real applications. Therefore, there have been great challenges for researchers to maintain high PLQY with improved photostability^15^ (overcoming the photodegradation) of colloidal quantum dots over a long period under ambient conditions.

To date, different strategies have been reported to overcome the above-mentioned problems, among which growing an inorganic outer shell with wider bandgap materials on the surface of colloidal quantum dots is found to be the most prominent one.^16^ The epitaxially grown inorganic shell materials are characteristically robust and provide physical and chemical stability to the core QD structure.^2,17–20^ During the inorganic shell growth (wider bandgap materials than CdSe) the lattice parameters of the core and shell need to be considered.^21^ The significant lattice mismatch between the core and shell could create interfacial lattice strain which leads to defects. CdSe core and CdS shell are some of the most studied systems due to the lowest lattice strain (lattice mismatch: 3.9%) with quasi-type-II band offsets.^22,23^ Growing thick or even “giant” shells is found to be a viable solution to enhance environmental stability or to suppress non-radiative processes associated with fluorescence intermittency (blinking) and photobleaching.^24^ CdS has been extensively used as the shell material, capable of much thicker (size around ∼20 nm) epitaxially grown shells that can afford suppressed blinking and photobleaching,^25^ with near-unity quantum yield,^26^ enabling this material for several applications, such as single-photon sources, molecular probes for single-particle tracking,^27^ and active emitters for low-threshold lasers^28^ and robust solid-state lighting.^29,30^

Due to the quasi-type-II band alignment in CdSe/CdS QDs, the non-blinking, photostable core/shell QDs are limited to providing emission in the red region of the visible spectrum (>600 nm).^25^ Upon photoexcitation, the hole is restricted to the CdSe core while the electronic wave function is extended into the shell, which results in a redshift in the emission. By employing a ZnS shell (which forms type I alignment) it is possible to obtain the entire visible range such as the blue- or green-color emission. But the huge (∼12%) lattice mismatch between ZnS and CdSe limits the achievable defect-free shell thickness. Because of this, graded alloy cores and/or shells have been utilized to avoid having an abrupt and large structural mismatch at the interface.^31^ Non-blinking, photostable green-emitting alloy core/alloy shell (CdZnSSe/ZnSSe) with a thick and giant ZnS SILAR (Successive Ionic Layer Adsorption and Reaction) shell has been reported recently.^31^ Despite the recent exploration of blinking properties of green-emitting giant quantum dots, relatively less is known about the details of photophysical properties such as the shell thickness-dependent photostability under continuous UV irradiation and the effect of temperature on the PLQY.^32,33^

Herein, we sought to gain access to bright and stable emission in the green region of the visible spectrum, with a particular interest of studying shell thickness-dependent photostability under continuous UV irradiation, and temperature-dependent PL properties of the ensemble nanocrystals. To study these we have synthesized different sized green-emitting giant core–shell or multilayer quantum dots through ‘Composition Gradient Synthesis’^34^ by using the ‘hot injection method as well as the SILAR process within a ‘single-pot’. We investigated their detailed optical properties, crystal structures, and size-dependent lifetime. Our studies concluded that upon reaching the critical thickness of the shell (core/shell diameter ∼ 8.5 nm) effective reduction of the surface-related recombination and improvement of the photostability of the QDs which were irradiated under strong UV light (8 W cm^−2^, 365 nm) can be achieved. It was found that shell growth beyond the critical size (beyond diameter ∼8.5 nm) does not have any further effect on photostability. QDs with particle size ≥ 8.5 nm retain ≥50% of photoluminescence intensity whereas sample-1 (size ∼7.12 nm) shows a drastic reduction in PL intensity and retains only <1% of its original value after 48 h of continuous UV irradiation. Furthermore, the photoluminescence (PL) spectra of these four different core/shell QDs were studied in the temperature range from 10 °C to 90 °C to understand the temperature effect on the radiative processes. A similar observation was found in the case of temperature-dependent PL properties, where the shell thickness plays a minimal role above diameter ≥ 8.5 nm. It is interesting to note that, the PLQY is highest when the size of the particle reaches 10.7 nm, and further growth of the ZnS shell *via* the SILAR process does not help in further improvement of the PLQY. The PLQY of QDs was dramatically increased from 22.5% to 62.8% after interfacing the CdSe core with gradient alloy shells with wider bandgap materials. Thus, we envisioned that these highly efficient green-emitting graded alloy core/shell QDs could be potentially utilized for LED applications as an efficient green emitter.

## Results and discussion

**Fig. 1:**
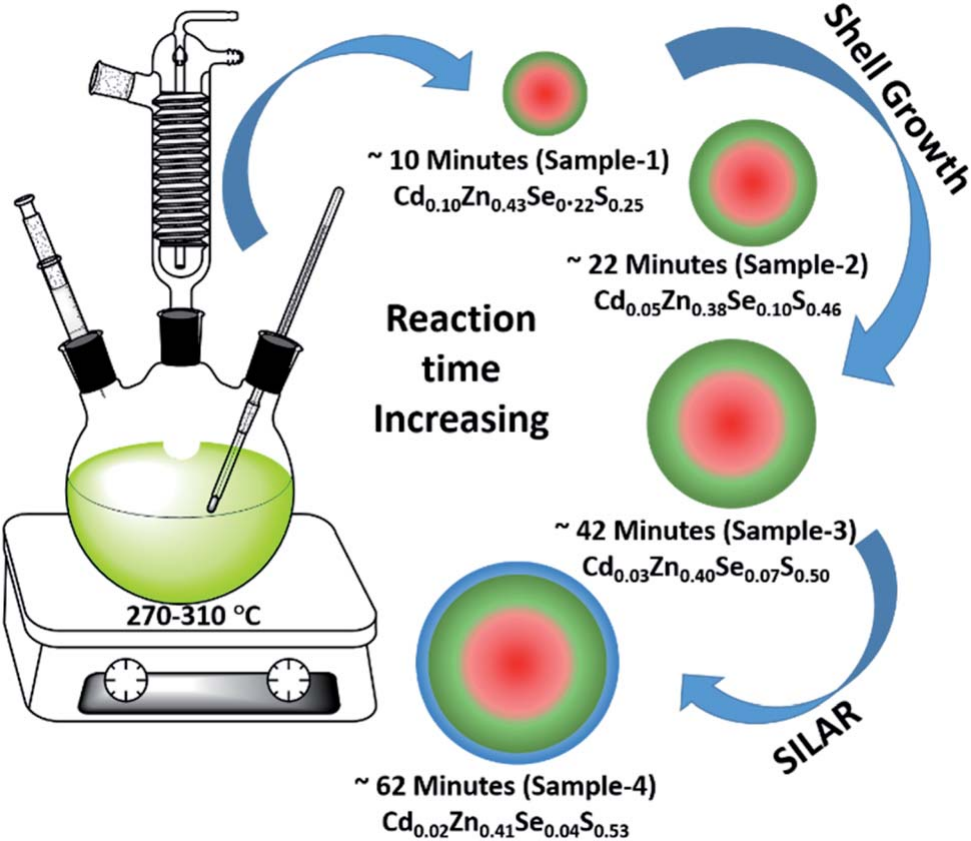
Schematic illustration of the synthesis of green-emitting QDs using gradient chemical composition synthesis method.

Using the gradient chemical composition synthesis method, highly emissive green-emitting QDs with four different shell thicknesses were prepared according to a previously reported method using four different precursors (Cd-OA, Zn-OA, TOP-Se, and TOP-S).^31,35^ The cationic precursors of Cd and Zn with a stoichiometric ratio of 1 : 24 were treated with a mixture of chalcogenides Se and S with an equivalent ratio at a reaction temperature of 310 °C. The details of the synthesis process are provided in the ESI.† According to the reactivity order, as soon as the mixture of S + Se is injected in the Cd + Zn precursor solution at 310 °C, initial nucleation of the CdSe core takes place with simultaneous formation of gradient alloy shells. Due to the presence of excess Zn and S precursors in the reaction medium, the outermost shell predominately formed a pure ZnS. The compositional evaluation over time is illustrated schematically in Fig. 1. Zn^2+^ is a hard Lewis acid compared to Cd^2+^, and thus the Zn–oleate complex is more stable than Cd–oleate.^36^ Similarly, the TOP–S complex is more stable than TOP-Se. Because of these reactivity differences, it is obvious that Cd and Se precursors react faster to undergo CdSe nucleation. Over time, Zn and S react slowly and allow the compositionally gradient shell growth.^37^ The evaluation of gradient alloy shell growth on the CdSe core of these green-emitting quantum dots was investigated with temporal changes by taking aliquots at different reaction time intervals. The different time interval samples (with different shell thicknesses) were characterized for the size, crystal structure, and photophysical properties of QDs.

**Fig. 2:**
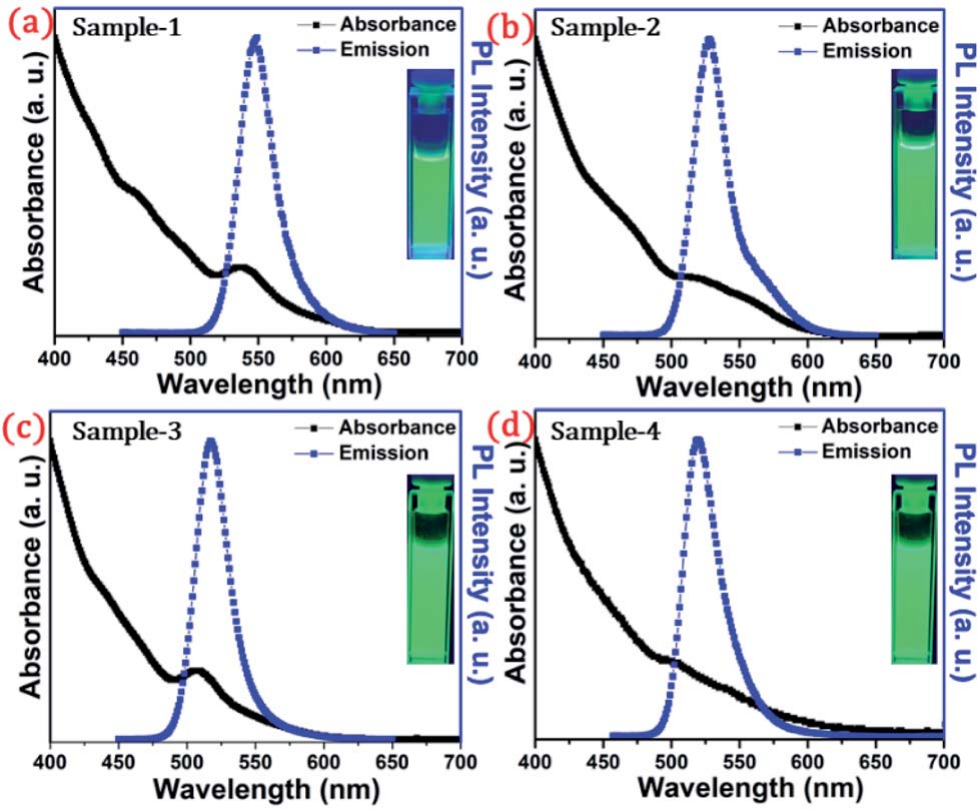
Absorbance (blackline) and PL intensity (redline) of the synthesized QDs dispersed in hexane (a) sample-1, (b) sample-2, (c) sample-3, and (d) sample-4. Inset shows the digital photographs of colloidal solution taken under UV light (*λ*_ex_ = 365 nm) irradiation.

**Fig. 3:**
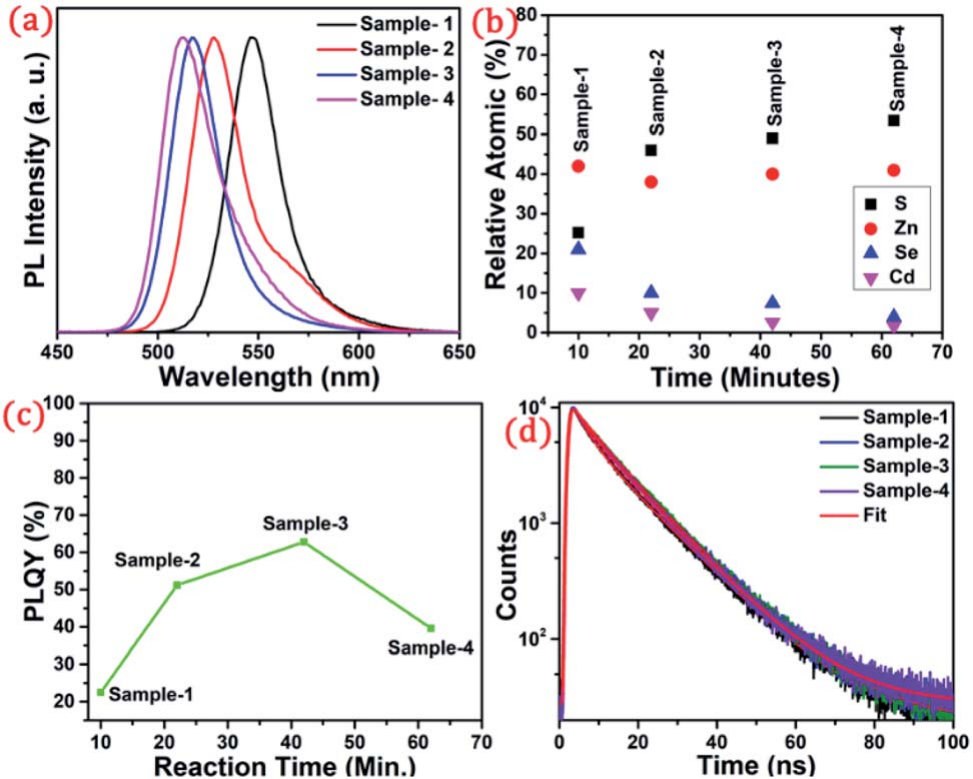
(a) PL spectra showing a blue shift from wavelength 547 to 512 nm. (b) Atomic percentage with increasing reaction time (c) change in the PLQY with respect to reaction time (d) time-resolved photoluminescence decay profiles of sample-1 to sample-4.

**Fig. 4:**
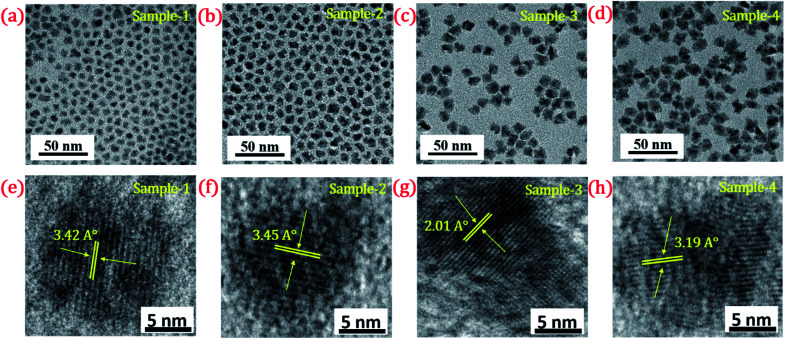
TEM image of (a) sample-1 (b) sample-2 (c) sample-3 (d) sample-4. HR-TEM images of (e–h) sample-1 to sample-4 with a scale bar of 5 nm. Inset shows their d-spacing values (°A).

The detailed optical characterization of the four green-emitting graded alloy shell QDs was performed using UV-Visible absorption and photoluminescence (PL) emission spectra, which can be seen in Fig. 2. The absorption and emission spectra with the 1st excitonic peak at 537 nm and the emission maximum at 547 nm are presented in Fig. 2(a) for the sample grown for 10 min. The full width at half maximum (FWHM) is 30.50 nm which indicates that they are moderately monodisperse particles. The samples grown for 22 min and 42 min and SILAR ZnS exhibit absorption peaks at 520 nm, 513 nm, and 503 nm and PL emission peaks at 528 nm (FWHM = 33.01 nm), 518 nm (FWHM = 28.41), and 512 nm (FWHM = 35.24 nm) respectively. The aliquots were collected at the early stage of the reaction (30 s) and presumably the core CdSe showed first excitonic absorption and emission maximum at 541 nm and 555 nm respectively (see Fig. S5†). Fig. 2(a–d) shows the absorption and emission of four different-sized green-emitting giant dots. All four samples are highly emissive which is evident from the digital image of the colloidal solution of synthesized green-emitting dots under UV irradiation (excitation at 365 nm) as depicted in the inset of Fig. 2(a–d). In the case of giant-shell CdSe/CdS QDs, a drastic redshift during the CdS shell growth is observed due to the leakage of the electronic wavefunction from the core to CdS shell. On the contrary, these graded alloy core/shell QDs exhibited a significant blue shift in emission during the shell growth (547 nm to 512 nm),^31^ enabling very large diameter green-emitting nanocrystals, as can be seen in Fig. 3(a). EDX spectra of all four as-synthesized quantum dot samples were collected to quantify the amount of each element present in the QDs. The relative atomic percentage with reaction time is shown in Fig. 3(b) and the detailed EDX spectrum can be seen in the ESI (Tables T6 to T9†). The EDX results presented in Fig. 3(b) show that sample-1 (10 min) is composed of 10.52% cadmium, 21.55% selenium, 25.22%, sulfur, and 42.71% zinc atoms. These results reflect that the sample-1 (10 min) structure has more Se than Cd atoms and more Zn than S atoms (see Fig. S1†). In sample-2, atomic concentrations of S, Zn, Se and Cd are 46.39%, 38.14%, 10.26% and 5.20% respectively (see Fig. S2†), and similarly, in sample-3, the atomic concentration of S, Zn, Se and Cd is 49.54%, 40.44%, 7.40% and 2.61% respectively (see Fig. S3†). In sample 4, the atomic concentrations of S, Zn, Se and Cd are 53.45%, 40.93%, 3.92% and 1.70% respectively (see Fig. S4†). It is evident from Fig. 3(b) that the atomic percentage of Cd and Se is high in sample-1 but it decreases when the reaction growth time increased from sample-1 to sample-4. On the other hand, the concentration of S increased with increasing the reaction time till 62 minutes. This implies that upon increasing the growth time the outer shell continued to grow which is predominately ZnS in nature. The blue shift is anticipated due to the continued alloying during the growth and incorporation of Zn and S into the core crystals throughout growth. During the growth of an additional ZnS shell *via* the SILAR process, the absorption and emission progressively shifted to further higher energies, even broadening the emission spectrum (FWHM ∼ 35.24). It is hypothesized that during the growth process at high temperature the intra-diffusion of Zn cations or S anions from the shell into the core occurs, resulting in an increment of the energy bandgap of band-edge luminescence. This intra-diffusion process eventually decreases the effective size of the CdSe core, leading to the blue shift.^36,38–41^ The calculated photoluminescence quantum yield (PLQY) values of the four samples are 22.5% (10 min), 51.2% (22 min), 62.8% (42 min), and 39.6% (SILAR-ZnS). The growth time-dependent (size dependent) PLQY of QDs was monitored and calculated by comparing with a standard organic dye, as shown in Fig. 3(c). At the reaction time of 10 min, the QDs exhibited a relatively low PLQY of 22.5%. However, after 42 min of growth, the PLQY was dramatically enhanced up to 62.8% due to the formation of Zn-based alloy shells, which can effectively confine the exciton wave function in the core due to the tunnelling effect. In a gradient alloy structure upon photoexcitation, the excitation can be transferred from outer shells into the inner core. In this graded alloy core/shell synthesis, the outermost shell is pure ZnS with a gradient intermittent shell, which electronically blocks the tunnelling of the excitons to the shell and effectively tunnels excitons from shells into the core. Due to the chemical composition variation in the gradient nature of the shell, the lattice mismatch and interfacial strain between core/shell are not that prominent, and thus discrete interfacial separation is not visible.^42^ As shown in the TEM image (Fig. 4(d)), a further thicker ZnS shell grew *via* the SILAR, yielding particles with non-homogeneity of the ZnS outer shell. This significant coarsening of the shape upon SILAR ZnS shell growth results in defects on the surface and thus a decrease in PLQY.

To understand the optical properties of the different-sized green-emitting giant QDs, we have studied the time-resolved PL of the four samples using the time-correlated single-photon counting (TCSPC) fluorescence technique. A prominent influence of the shell size on the PL decay behaviour of the graded alloy core/shell is observed (Table T1†). The time-resolved PL decay profiles of the four samples are shown in Fig. 3(d) and the analysed decay components are tabulated in Table T1 in the ESI.† As the size of the gradient alloy shells increases, the PL lifetime is gradually increased (from sample-1 to sample-3) from 8.44 ns to 10.43 ns and 10.61 ns, which infers that the nonradiative decay channel is largely suppressed and thus electron–hole pairs can live longer in a gradient structure, which eventually undergoes radiative recombination.^43^ The formation of gradient alloy shells makes exciton transfer toward the core, causing a substantial increase of the radiative recombination rate relative to nonradiative recombination with a significant enhancement in the PL lifetime. As the graded alloy shell continues to grow on the core, the smooth epitaxial growth process facilitates more the radiative recombination process and constrains the nonradiative recombination process. It should be noted upon additional ZnS SILAR growth there are not many changes in the PL lifetime (sample-4, lifetime-9.44 ns). The radiative and non-radiative rate constants (*k*_r_ and *k*_nr_, respectively), estimated from the measured PLQY and *τ*_Avg_ values, show that the *k*_nr_ value decreases upon shell growth (Table T1†) indicating a significantly suppressed non-radiative recombination and high PLQY of the systems.

**Fig. 5:**
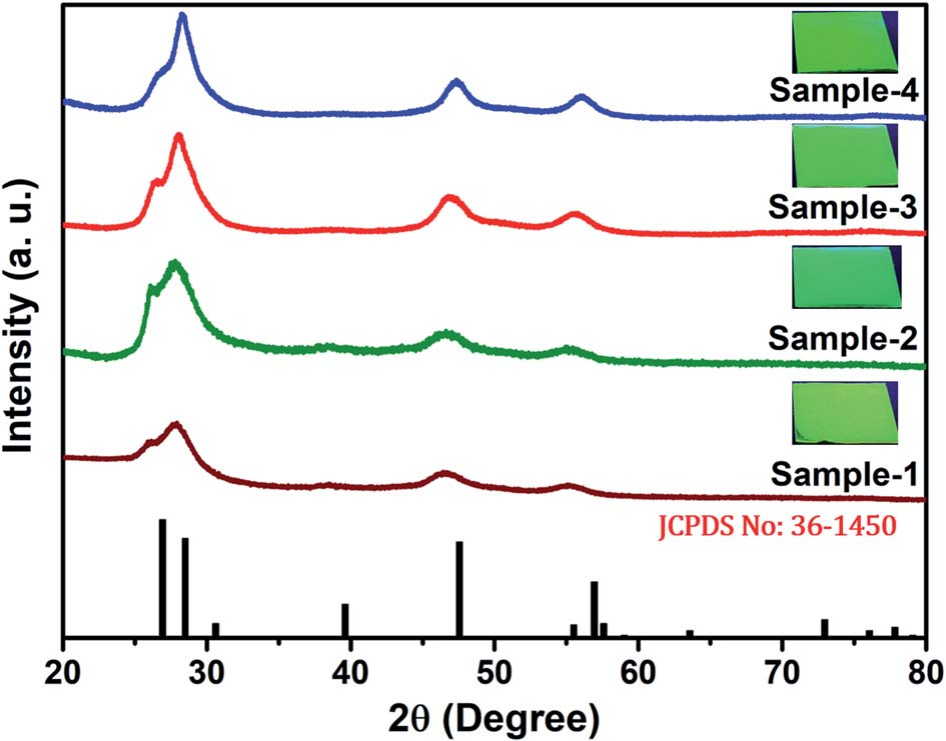
XRD pattern of the synthesized QDs at various reaction times of sample-1 to sample-4 (bottom to top) respectively. Inset shows the digital images of thin films taken under UV light (*λ*_ex_ = 365 nm) irradiation.

The size and shape of all four samples were measured using TEM, and are shown in Fig. 4(a–d). The size of nanocrystals was increased from 7.12 to 10.70 nm by increasing the gradient alloy shell thickness over the growth time. The particle size distribution histograms of all four samples are shown in Fig. S6.†Fig. 4(e–h) shows the high-resolution transmission electron microscopy images of a single nanocrystal with a scale bar of 5 nm which confirms the presence of defined lattice fringes, suggesting the high crystallinity of all four samples. The calculated d-spacing of the four samples is 3.42, 3.45, 2.01, and 3.19 A° which corresponds to the (100), (100), (110), and (002) plane of the hexagonal phase, respectively. The TEM image of the 10 min sample in Fig. 4(a) shows that the NCs have a spherical morphology with an average particle size of 7.12 ± 1.2 nm. Fig. 4(b–d) present the transmission electron microscopy (TEM) images of 22, 42 and SILAR samples and the calculated average particle size of the samples is 8.50 ± 1.3 nm, 10.70 ± 2.1 nm, and 12.21 ± 2.1 nm respectively. All four samples synthesized with different growth times show quasi-spherical shapes with a narrow size distribution with highly improved crystallinity. As shown in the HRTEM image, there was no discrete interfacial layer evident between the core and shells for the synthesized QDs which infers smooth epitaxial growth and a gradient chemical composition change from the core to ZnS shell. The crystallographic phases of the four different samples were investigated using powder X-ray diffraction (XRD) as shown in Fig. 5. All four XRD diffraction patterns were well matched with the hexagonal crystal phase of ZnS, matching with the previously reported pattern (JCPDS file no. 36-1450). The XRD patterns of the QDs with composition gradients were obtained at 10 min, 22 min, 42 min, and SILAR (denoted as sample-1, sample-2, sample-3, and sample-4, respectively). The XRD patterns of bulk hexagonal ZnS are also shown at the bottom in Fig. 5. The thin films were prepared by drop-casting the four different colloidal solutions onto a clean glass slide and their images under UV irradiation are shown in the inset of Fig. 5.

**Fig. 6:**
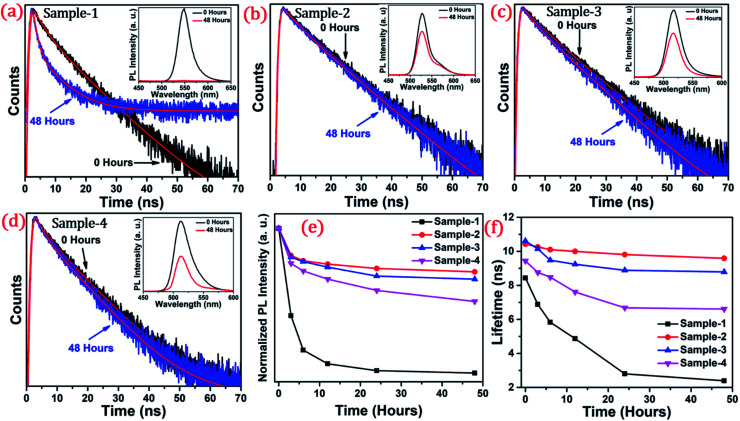
The PL decay curve of graded alloy green-emitting QDs after the irradiation time of 0 hours and 48 hours of (a) sample-1 (b) sample-2 (c) sample-3 and (d) sample-4. Inset shows the PL spectra of the samples after 0 hours (black line) and 48 hours (red line) (e) normalized PL intensity and (f) the lifetime in ns of sample-1 to sample-4 with increasing irradiation time.

The long-term stability of quantum dot-based devices is probably one of the major issues for wide-scale applications of these green-emitting QDs. The photostability of these QDs is one of the major hurdles for practical utilization. The QDs with greater than 8.5 nm size are found to be much more stable under different external conditions. We studied the photostability of the four different-sized core–shell giant quantum dots. At regular time intervals, the PL intensities were measured for each sample at room temperature, under continuous illumination of UV light (365 nm, 8 W cm^−2^) for 48 hours. Simultaneously, we have also studied and measured the time-resolved PL for each sample which was under continuous illumination of UV light for 48 hours. The changes in the lifetime decay profile and the PL intensities (in inset) under continuous strong UV irradiation after 0 and 48 hours are shown in Fig. 6(a–d). The PL decay profiles of the samples with prolonged UV light exposure are presented in Fig. 6(a–d). The PL decay curves are well-fitted with the bi-exponential function when the sample is UV exposed up to 6 hours. The two lifetime components are ascribed to band-edge recombination and shallow trap recombination.^44,45^ At 0 hours, the decay trace of sample-1 (see Table T2 in ESI†) is fitted with lifetime components of 3.54 ns (*τ*_2_) and 12.29 ns (*τ*_3_) which are assigned to band edge recombination and shallow trap recombination respectively. Upon prolonged exposure, (after continuous UV irradiation) the bi-exponential decay traces are transformed into tri-exponential decay with the additional lifetime component (*τ*_1_) attributed to deep traps.^44,45^ In this case, with prolonged exposure to UV light for 12 hours, the sample exhibited tri-exponential decay profiles with lifetime components 0.82 ns (*τ*_1_), 4.22 ns (*τ*_2_), and 11.7 ns (*τ*_3_) which are ascribed to deep traps, band-edge recombination, and shallow traps respectively. Moreover, it is observed that the lifetime component attributed to band-edge recombination (*τ*_2_) and shallow-trap recombination (*τ*_3_) is gradually reduced. The deep traps become predominant with prolonged UV exposure as inferred from the increment of *α*_1_. Unlike sample-1 and sample-4, the decay traces of sample-2 (see Table T3 in ESI†) and sample-3 (see Table T4 in ESI†) are well-fitted with the biexponential function indicating the absence of deep traps. The lifetime components remain unchanged with the continuous UV irradiation for 48 hours. Unambiguously, the PL lifetime parameters explain the excellent photo-stability of sample-2 and sample-3.

The photoluminescence intensity is decreased dramatically for sample-1 and it almost quenched completely within 24 hours as seen in Fig. 6(a). The decrease in the PL intensity is attributed to the thin outer graded alloy shell around the core which is relatively small in diameter. Thus, oxidation happens on the surface or at the interface between the CdSe core and graded alloy shell during UV irradiation. The PL intensities of sample-2 and sample-3 at 0 and 48 hours of continuous UV irradiation shown in Fig. 6(b and c) (in inset) depict the high stability due to increased shell thickness. Sample-1 retains only <1% of the PL intensity whereas sample-2, -3 and -4 QDs retain ∼70.30%, 65.27%, and 50.02% of their initial PL intensity under continuous illumination of UV light (365 nm, 8 W cm^−2^) after 48 h. Generally, it's notable that the UV-induced PL intensity is vastly maintained with the increase of the ZnS shell diameter, and the thickness of the ZnS shell is strongly associated with photobleaching. With the continuous alloy graded shell growth on the core, the surface trap is reduced due to smooth epitaxy graded alloy shell with reduced lattice strain. Moreover, the large diameter shell obstructs the oxygen diffusion into the core, which can reduce surface oxidation. Sample-4 which is synthesized with the SILAR process and showing non-homogeneity shell growth produces some surface defects and thus a decrease in PLQY was observed, but the stability under UV irradiation is mostly maintained. Fig. 6(e) presents the trend of normalized PL intensity with time in hours and Fig. 6(f) presents the PL lifetime variations of the four samples with time in hours under continuous UV irradiation. In both figures, the trend of PL intensity and lifetime goes down after 48 hours for sample-1, but, when the shell thickness is increased around the core, there is less effect on the PL intensity as well as the lifetime of sample-2 and sample-3. Fig. S7(a–d) in the ESI† show the changes in PL intensities of the four samples with time. Fig. S8(a–d)† show the time-resolved PL spectra of sample-1 to sample-4 respectively at different time intervals. PL intensity and lifetime are recorded at regular time intervals when samples are exposed to continuous UV-light and the calculated lifetime parameters are well tabulated in Tables T2 to T5.† Fig. S9 in the ESI† shows no change in the PL wavelength during the photostability study under UV irradiation from 0 to 48 hours.

**Fig. 7:**
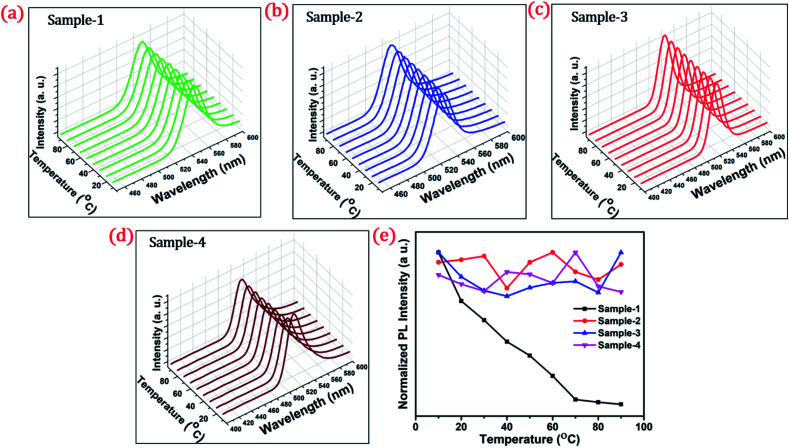
Temperature-dependent stability test of QDs at a different temperatures from 10 to 90 °C and the PL spectra of (a) sample-1 (b) sample-2 (c) sample-3 and (d) sample-4 respectively (e) normalized PL intensity of the four samples with temperature (°C).

To investigate the shell effect on the nonradiative relaxation processes in QDs, we analyse the temperature dependence of PL intensity. Fig. 7(a–d) show the temperature-dependent PL spectra of the four QDs recorded in the temperature range from 10 °C to 90 °C. The normalized PL intensities of all four samples with respect to temperature are shown in Fig. 7(e). The PL intensities of sample-1 are reduced which infers that there is a large amount of nonradiative traps on the surface of the core QDs, causing thermal quenching as also explained by TRPL. In contrast, the PL intensity of sample-2, -3 and -4 is significantly maintained with increasing temperature, as shown in Fig. 7(e). This means that there are minimum nonradiative traps on the surface of the QDs (for sample-2, -3 and -4), causing less thermal quenching. Generally, the PL QY and photostability of the core QDs can be improved by growing a shell with a larger bandgap to confine electrons and holes into the core. The surface traps that are responsible for nonradiative relaxation are efficiently passivated by the growth of a graded giant shell. It is also noted that (see Fig. S10†) the emission maximum of the four samples does not show any change with increasing temperature.

## Conclusions

In conclusion, highly efficient green-emitting graded alloy core/shell QDs have been synthesized using a hot colloidal method in a “one-pot” reaction. An additional ZnS shell was grown using Successive Ionic Layer Adsorption and Reaction (SILAR). We studied the shell thickness-dependent photostability of the colloidal solution under continuous UV irradiation, and upon varying the temperature from 10–90 °C. Our study concludes that the size of the QDs and to be specific, the shell thickness plays an important role in achieving highly photostable colloidal stable green-emitting giant quantum dots. The QDs with a diameter ≥ 8.5 nm show improved photostability under continuous UV light irradiation and with increasing temperature. Thus, we can envision that green-emitting gradient alloy giant QDs with specific shell thickness and with superior photostability can be used for highly efficient green-emitters and potentially applicable for the fabrication of green LEDs.

## Author contributions

Rahul Singh conducted all syntheses, under the guidance of Nimai Mishra and collected all data with Syed Akhil. Rahul Singh and Syed Akhil analysed all data. Rahul Singh, Syed Akhil, V.G. Vasavi Dutt and Nimai Mishra wrote the manuscript.

## Conflicts of interest

There are no conflicts to declare.

## Supplementary Material

NA-003-D1NA00663K-s001
