# Supplementary material for: Shell thickness dependent photostability studies of green-emitting “Giant” quantum dots
Source: Nanoscale Adv. 2021 Sep 24;3(24):6984–91. doi: 10.1039/d1na00663k (PMC9417657; doi:10.1039/d1na00663k)
Supplement: NA-003-D1NA00663K-s001 [file NA-003-D1NA00663K-s001.pdf]

Electronic Supplementary Material (ESI) for Nanoscale Advances.
This journal is © The Royal Society of Chemistry 2021

Supporting Information

Shell Thickness Dependent Photostability Studies of Green-Emitting "Giant" Quantum Dots

Rahul Singh, Syed Akhil, V. G. Vasavi Dutt, Nimai Mishra*

Department of Chemistry, SRM University-AP, Andhra Pradesh, Neerukonda, Guntur (Dt), Andhra Pradesh, India 522240.

*Email ID: nimai.m@srmap.edu.in

Chemical and methods:

Sulphur Powder (S, 99%), selenium powder (Se, 99.5%) and zinc oxide (ZnO, 99%) were purchased from Finar. Cadmium acetate ($Cd(OAc)_2$, 98%) was purchased from Fisher scientific. Zinc acetate dihydrate ($Zn(OAc)_2$, 98%), 1-Octadecene (ODE, 90%) and Trioctylphosphine (TOP, 90%) were purchased from Sigma Aldrich. Oleic acid (OA, 90%) was purchased from Alfa Aesar. We used all the chemicals without any further purification. Moisture/air sensitive compounds were assigned with using standard Schlenk line techniques under an inert gas atmosphere.

Synthesis of Green- Emitting 'Giant' Quantum dots:

In synthesis of green- emitting core/ shell gradient alloy QDs,

Single-step or one-pot synthesis is a typical synthetic procedure to obtain the colloidal quantum dots. In a three neck 50 ml round bottom flask 3.41 mmol of ZnO, 0.14 mmol of $Cd(OAc)_2$ were mixed gently with 7 ml of OA at 150 °C with purging nitrogen gas in the RB flask. After reaching the temperature of the mixture at 150 °C, 15 ml ODE was also injected. After adding the ODE, the temperature of the mixture was heated to 310 °C.

At the same time, we also prepared the 4 vials of different anionic stock solutions. After reaching the temperature of RB flask at 310 °C, a solution of 5 mmol of S and 5 mmol of Se in 5 ml of TOP swiftly injected into the RB flask at 10 min the aliquot was collected. After 10 minutes, the growth of CdSe/ZnS quantum dots, at the same temperature a stock solution of 1.6 mmol of S which was dissolved in 2.4 ml of ODE again injected for obtaining the uniquely large size CdSe/ZnS/ZnS QDs through the ZnS overcoating then hold for 12 min. In the same continuation, a zinc stock solution prepared with 2.86 mmol of $Zn(OAc)_2$ which was dissolved in the 4 ml of ODE and 1 ml OA injected in the RB flask at 310 °C. After injection the temperature lower down and then the temperature was set at 270 °C, a S stock solution

which prepared by 9.65 mmol of S in 5 ml of TOP injected dropwise in the RB flask with the rate of 0.5ml/min to form a thick ZnS shell. This is called SILAR process in which the solution is injected in the portions with dropwise.[1,2] After completing the injection, the growth of quantum dots was continuing at 270 °C for 20 min. The resulting CdSe/ZnS and CdSe/ZnS/ZnS colloidal quantum dots were precipitated in the ethanol and washed with solvent combination ethanol-hexane mixture in the ratio of 1:4 by centrifugation on 12000 rpm. Washed and purified quantum dots were dispersed in the hexane for further analysis and characterization.

Characterization of Green- Emitting 'Giant' Quantum dots:

UV-Vis absorption and PL measurements

CdSe/ZnS 'Giant' QDs and aliquots were dispersed in hexane with absorbance scanning mode UV-Visible spectra were collected in UV-Visible spectrophotometer UV-2600i Shimadzu. Photoluminescence or PL spectra of CdSe/ZnS 'Giant' QDs and aliquots were recorded with FlouroLog-3 (Horiba Jobin Yvon).

PL measurements and PLQY

Dye Cumarine 153 was used as a reference in ethanol (QY=0.546) for the measure of photoluminescence quantum yield (PLQY). For the measurements of PLQY, we have matched optical density 0.17 at 420 nm wavelength. The identical instrumental analysis were used to measure the areas under the fluorescence spectra curve.

Powder X-ray diffraction measurements

Green emitting 'Giant' QDs were dropped cast on the surface of neat and clean glass slides for powder X-ray diffraction which were measured with Empyrean PANalytical X-Ray Diffractometer with Cu-Kα X-radiation (λ = 1.5406 Å) at 40 kV and 30 mA power.

Transmission electron microscope (TEM) analysis

Transmission electron microscope (TEM) was used to characterization of size, distribution size, and structure of CdSe/ZnS QDs and the sample prepared by drop of an optimum solution of CdSe/ZnS QDs in hexane on copper (Cu) grid coated with carbon film using JEOL JEM-2100 High-Resolution Transmission Electron Microscope with 0.23 nm point resolution.

Time resolved photoluminescence (TRPL) analysis

Time resolved photoluminescence was measured by time-correlated single photon counting by TCSPC spectrometer (Horiba Jobin Yvon IBH) with laser diode at 372 nm excitation laser source and PL decay fitted and analysis by IBH DAS6 software.

Photostability measurements

Photostability was measured by a photoluminescence spectrometer (Horiba Jobin Yvon IBH) in the hexane. All four samples were kept for 48 hours under continuous UV irradiation with 365 nm of excitation at power density of 8 W/cm^2 then the spectra was recorded with photoluminescence (PL) intensity and time-resolved photoluminescence (TRPL) were taken after different time intervals at 0, 3, 6, 12, 24, and 48 hours.

Temperature dependent stability measurements

For the temperature dependent PL spectra, the samples were dispersed in toluene. Then temperature-dependent PL spectra of four QDs were recorded with Horiba Jobin Yvon IBH using with TC1 temperature controller Quantum northwest along with koolance with100-240 VAC and temperature from 10 °C to 90 °C.

Time resolved photoluminescence (TRPL) decay parameters analysis

The photoluminescence decay parameters of the as-synthesized and treated CdSe/ZnS 'giant' quantum dots and calculated radiative (k_r) and non-radiative (k_{nr}) rate constant. The lifetimes are calculated in nanosecond (ns).

PL lifetime decay parameters of sample-1 to sample-4. PL lifetime, $K_{r,}$ and K_{nr} values were calculated as per below equations.

$$\tau_{Avg} = \Sigma\tau i\alpha i/\Sigma\alpha_i,$$

$$K_r = PLQY/\tau_{Avg} \text{ and}$$

$$K_{nr} = (1 - PLQY)/\tau_{Avg}.$$

α_i is the amplitude which is associated with i^{th} lifetime constituent.

Table-T1: PL lifetime decay parameters with K_r and K_{nr} values of four samples

Sample	α_1	τ_1 (ns)	α_2	τ_2 (ns)	PLQY (%)	τ_{Avg} (ns)	K_r/ns^{-1}	K_{nr}/ns^{-1}
1-Sample	0.44	3.54	0.56	12.29	22.6	8.44	0.0266	0.0918
2-Sample	0.27	4.98	0.73	12.45	51.2	10.43	0.0490	0.0467
3-Sample	0.30	5.88	0.70	12.64	62.8	10.61	0.0591	0.0350

4-Sample	0.33	4.34	0.67	11.95	39.6	9.44	0.0419	0.0633

Table-T2: PL lifetime decay parameters of sample-1 under UV irradiation

Sample-1 (Hours)	α_1	τ_1 (ns)	α_2	τ_2 (ns)	α_3	τ_3 (ns)	τ_{Avg} (ns)
0			0.44	3.54	0.56	12.29	8.44
3			0.53	2.81	0.47	11.48	6.88
6			0.60	2.38	0.4	11.01	5.83
12	0.58	0.82	0.28	4.22	0.14	11.77	3.305
24	0.66	0.74	0.24	3.52	0.1	10.79	2.80
48	0.69	0.60	0.23	2.79	0.07	10.69	2.39

Table-T3: PL lifetime decay parameters of sample-2 under UV irradiation

Sample-2 (Hours)	α_1	τ_1 (ns)	α_2	τ_2 (ns)	τ_{Avg} (ns)
0	0.27	4.98	0.73	12.45	10.43
3	0.28	4.89	0.72	12.36	10.26
6	0.30	4.87	0.70	12.35	10.10
12	0.31	4.85	0.69	12.32	10.00

24	0.33	4.80	0.67	12.29	9.81
48	0.35	4.75	0.65	12.21	9.59

Table-T4: PL lifetime decay parameters of sample-3 under UV irradiation

Sample-3 (Hours)	α_1	τ_1 (ns)	α_2	τ_2 (ns)	τ_{Avg} (ns)
0	0.30	5.88	0.70	12.64	10.61
3	0.31	5.40	0.69	12.27	10.14
6	0.32	4.90	0.68	11.64	9.48
12	0.34	4.81	0.66	11.54	9.24
24	0.35	4.62	0.65	11.19	8.89
48	0.36	4.58	0.64	11.16	8.79

Table-T5: PL lifetime decay parameters of sample-4 under UV irradiation

Sample-4 (Hours)	α_1	τ_1 (ns)	α_2	τ_2 (ns)	α_3	T_3 (ns)	τ_{Avg} (ns)
0			0.33	4.34	0.67	11.95	9.44
3			0.34	3.49	0.66	11.48	8.76
6			0.36	3.33	0.64	11.38	8.48
12	0.22	1.47	0.39	6.17	0.39	12.51	7.61

24	0.31	0.91	0.34	6.36	0.35	12.10	6.68
48	0.25	0.68	0.31	4.52	0.44	11.46	6.61

Table-T6: EDX data of Sample-1

Element	Line Type	k Factor	k Factor type	Absorption Correction	Wt%	Wt% Sigma	Atomic %
S	K series	1.000		1.00	12.47	1.78	25.22
Zn	K series	1.277		1.00	43.05	3.17	42.71
Se	K series	1.683		1.00	26.24	2.74	21.55
Cd	L series	1.878		1.00	18.24	3.24	10.52
Total:					100.00		100.00

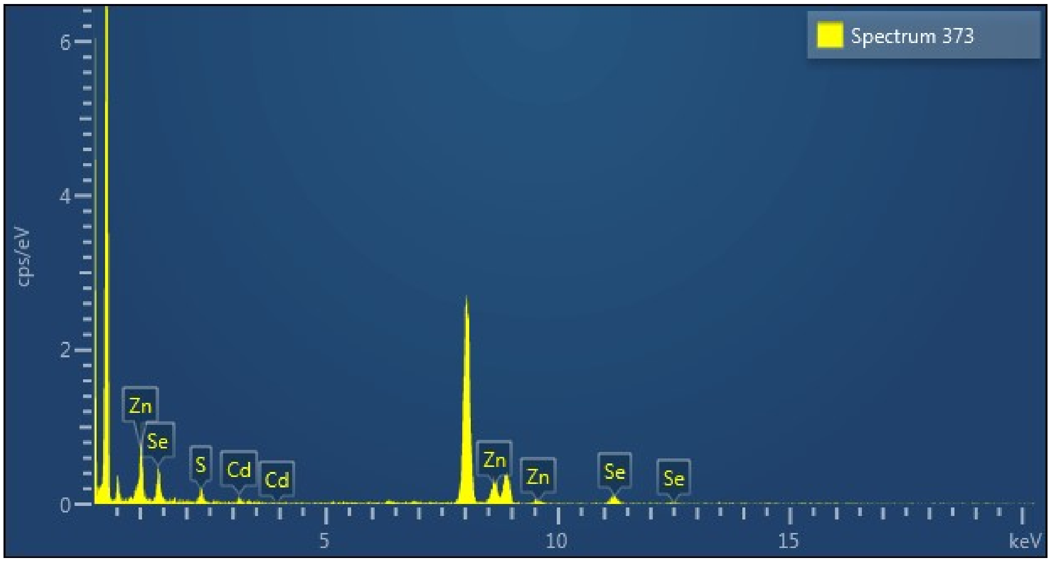

Figure-S1: EDX graph of Sample-1 showing atomic percentage

Table-T7: EDX data of Sample-2

Element	Line Type	k Factor	k Factor type	Absorption Correction	Wt%	Wt% Sigma	Atomic %
S	K series	1.000		1.00	27.67	1.98	46.39
Zn	K series	1.277		1.00	46.38	2.59	38.14
Se	K series	1.683		1.00	15.07	2.00	10.26
Cd	L series	1.878		1.00	10.87	2.62	5.20
Total:					100.00		100.00

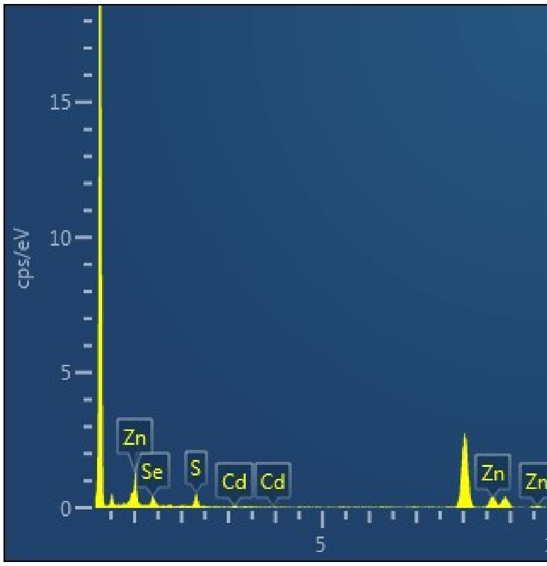

Figure-S2: EDX graph of Sample-2 showing atomic percentage

Table-T8: EDX data of Sample-3

Element	Line Type	k Factor	k Factor type	Absorption Correction	Wt%	Wt% Sigma	Atomic %
S	K series	1.000		1.00	31.08	0.95	49.54
Zn	K series	1.277		1.00	51.73	1.13	40.44
Se	K series	1.683		1.00	11.44	0.82	7.40
Cd	L series	1.878		1.00	5.74	0.95	2.61
Total:					100.00		100.00

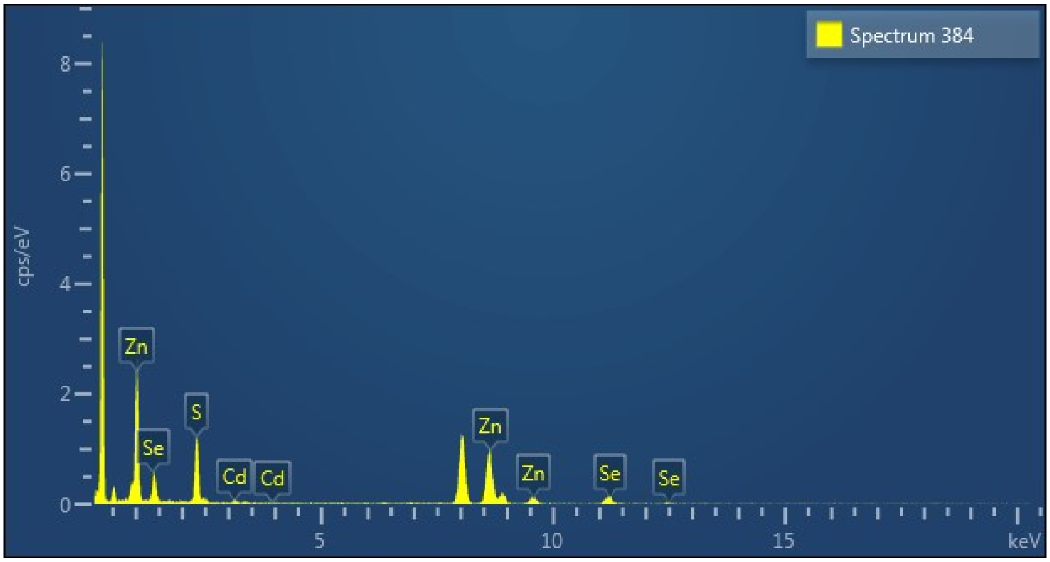

Figure-S3: EDX graph of Sample-3 showing atomic percentage

Table-T9: EDX data of Sample-4

Element	Line Type	k Factor	k Factor type	Absorption Correction	Wt%	Wt% Sigma	Atomic %
S	K series	1.000		1.00	35.05	1.12	53.45
Zn	K series	1.277		1.00	54.72	1.26	40.93
Se	K series	1.683		1.00	6.33	0.77	3.92
Cd	L series	1.878		1.00	3.91	0.97	1.70
Total:					100.00		100.00

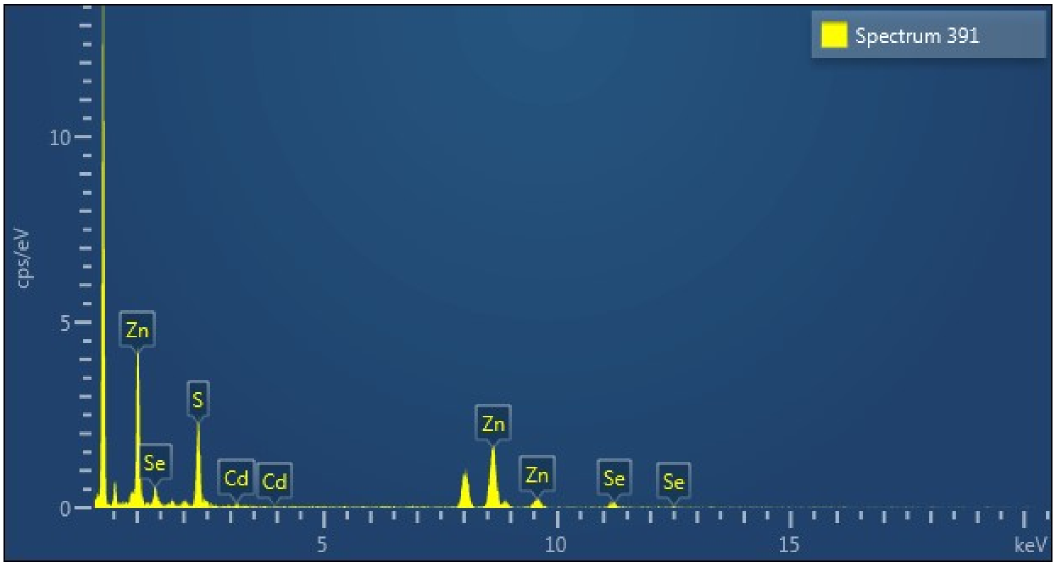

Figure-S4: EDX graph of Sample-4 showing atomic percentage

Table-T10: Photostability with previously reported are listed below

Material composition	Emission-PL_{max} (nm)<br>Average size (nm)	Photostability	References
$CdSe_xS_{1-x}/ZnSe_yS_{1-y}$	480 nm<br>1.5 to 8.5 nm	White halogen lamp (757 mW/cm) for 24 hours	Cho *et al.* 2017[3]
$CdSe_yZn_{1-x}S_{1-y}$	585 nm<br>11 to 9.6 nm	CW light (1.3 mW/cm^2 – 450 nm) for 3 hours	Chen *et al.* 2017[4]
InP@ZnSeS	522 nm<br>0.9 to 1.7 nm	UV irradiation (2 mW/cm^2 – 352 nm) for 24 hours	Lim *et al.* 2011[5]
InP/ZnS	4.3 nm<br>525 nm	Blue LED (34 W/m^2 – 468 nm) for 20 hours	Watanabe *et al.* 2018[6]
CdSe core/gradient alloy shell/shell (CGASS)	---<br>6.5 to 7.5 nm	UV lamp (8 W – 360 nm) for 24 hours	Roy *et al.* 2016[7]
In(Zn,Ga)P/ZnSeS/ZnS	532 nm<br>2.1 to 5.3 nm	UV-Light (800 $\mu W/cm^2$ – 365 nm) for 96 hours	Kim *et al.* 2019[8]
CdSe/CdS and CdSeS/ZnS	528 nm<br>3.9 to 13.1 nm	Violet lamp (60 W – 400) for 46 hours	Huang *et al.* 2016[9]

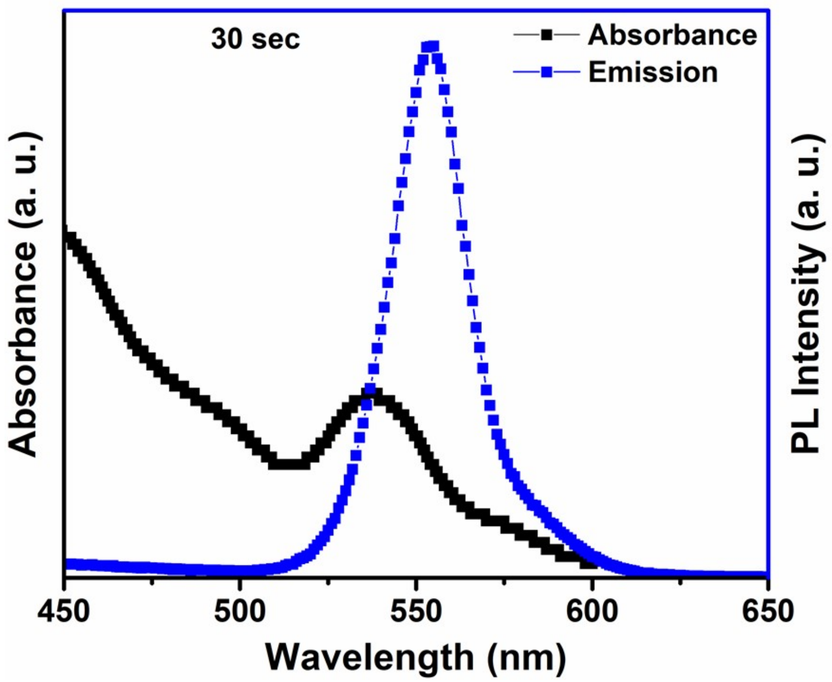

Figure-S5: Absorbance (Black line) and PL intensity (Blue line) spectra of aliquot at 30 sec reaction time

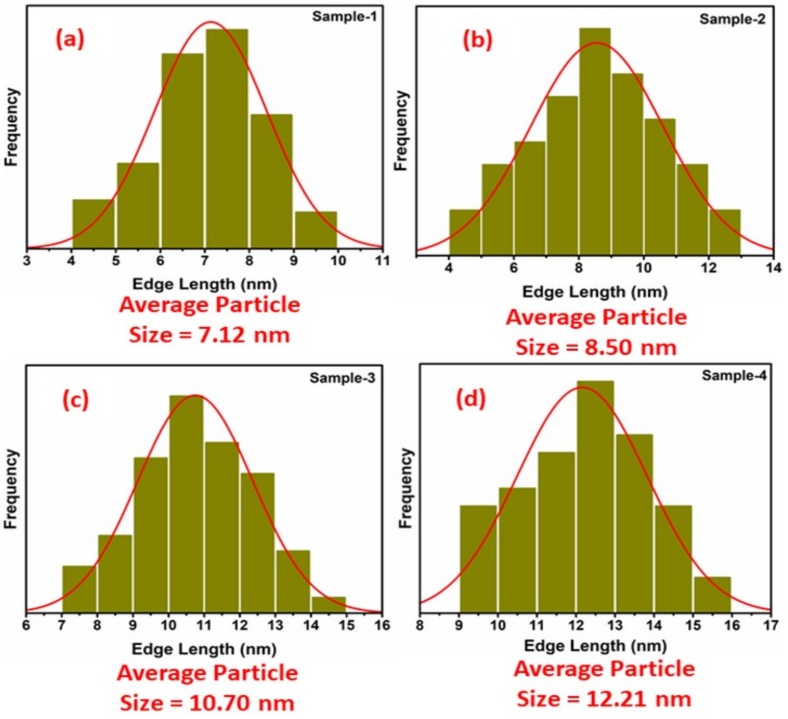

Figure-S6: Particle size distribution histograms of (a) Sample-1, (b) Sample-2, (c) Sample-3, and (d) Sample-4 with average particle size

Figure-S7: Photoluminescence intensity decay profiles of (a) Sample-1, (b) Sample-2, (c) Sample-3, and (d) Sample-4 under UV irradiation from 0 to 48 Hours.

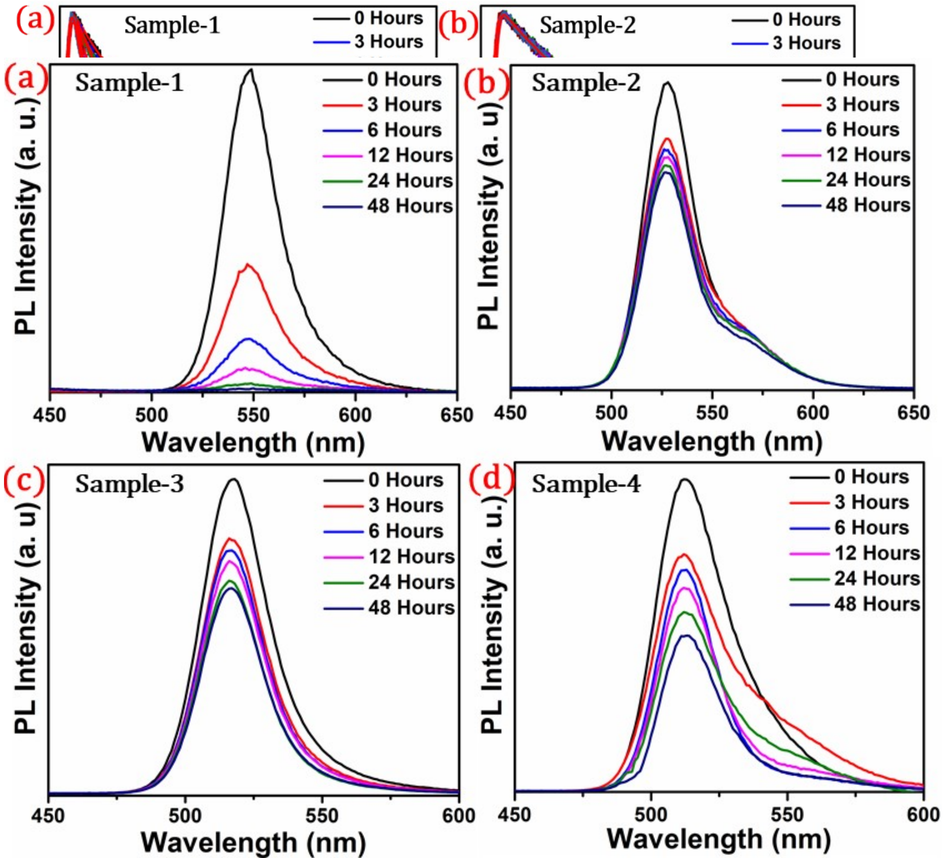

Figure-S8: Time resolved photoluminescence decay profiles of (a) Sample-1, (b) Sample-2, (c) Sample-3, and (d) Sample-4 under UV irradiation from 0 to 48 Hours

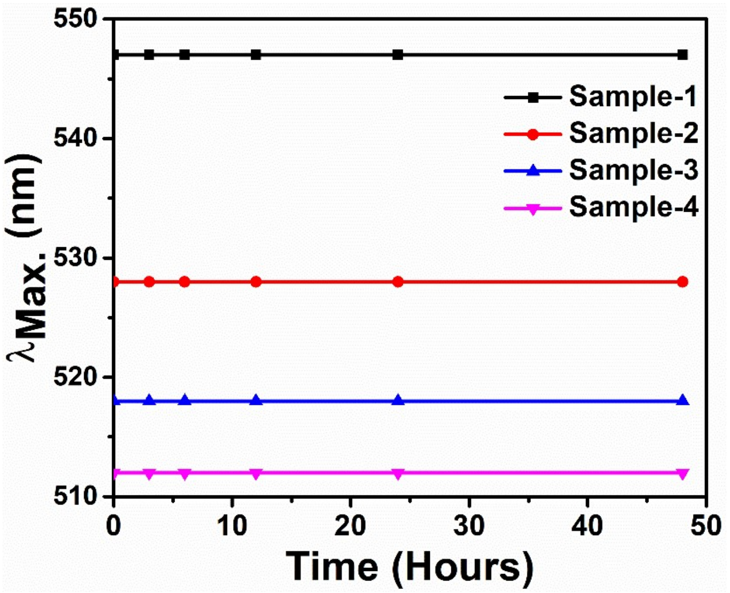

Figure-S9: Photostability of sample-1 to sample-4 their PL-Wavelength maximum (λ_{max}) with Time (Hours) under continuous UV irradiation from 0 to 48 hours.

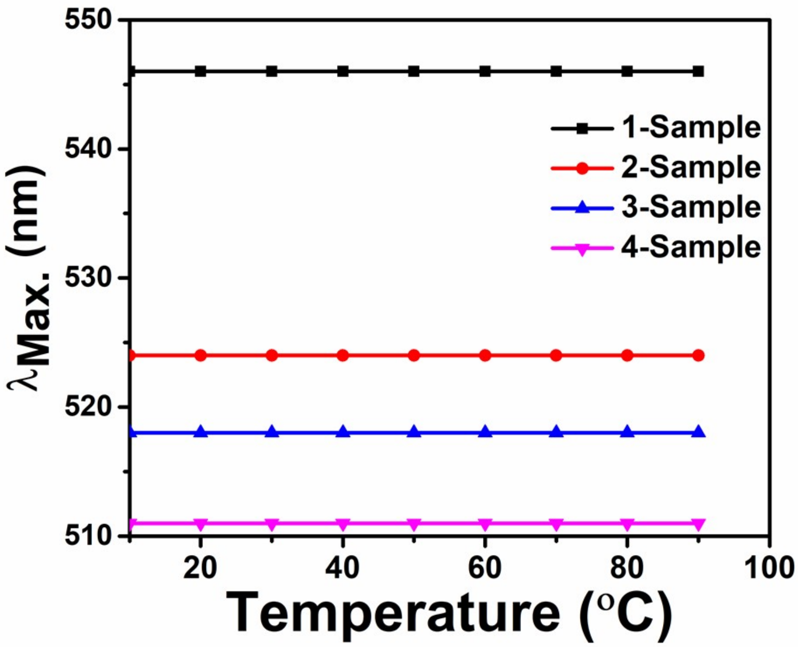

Figure-S10: Temperature dependent stability of sample-1 to sample-4 their PL-Wavelength maximum (λ_{max}) with temperature from 10 to 90°C.

References:

1 K.-H. Lee, J.-H. Lee, H.-D. Kang, B. Park, Y. Kwon, H. Ko, C. Lee, J. Lee and H. Yang, *ACS Nano*, 2014, **8**, 4893–4901.
2 J. R. McBride, N. Mishra, S. M. Click, N. J. Orfield, F. Wang, K. Acharya, M. F. Chisholm, H. Htoon, S. J. Rosenthal and J. A. Hollingsworth, *J. Chem. Phys.*, 2020, **152**, 124713.
3 J. Cho, Y. K. Jung, J.-K. Lee and H.-S. Jung, *Langmuir*, 2017, **33**, 3711–3719.
4 J. Chen, B. Yang, C. Li, K. Zheng, K. Žídek and T. Pullerits, *ACS Omega*, 2017, **2**, 1922–1929.
5 J. Lim, W. K. Bae, D. Lee, M. K. Nam, J. Jung, C. Lee, K. Char and S. Lee, *Chem. Mater.*, 2011, **23**, 4459–4463.
6 T. Watanabe, Y. Iso, T. Isobe and H. Sasaki, *RSC Adv.*, 2018, **8**, 25526–25533.
7 D. Roy, T. Routh, A. V. Asaithambi, S. Mandal and P. K. Mandal, *J. Phys. Chem. C*, 2016, **120**, 3483–3491.
8 M. Kim, W. H. Shin and J. Bang, *J. Lumin.*, 2019, **205**, 555–559.
9 B. Huang, R. Xu, N. Zhuo, L. Zhang, H. Wang, Y. Cui and J. Zhang, *Superlattices Microstruct.*, 2016, **91**, 201–207.
